# A Novel Intronic Variant in the KH3 Domain of HNRNPK Leads to a Mild Form of Au‐Kline Syndrome

**DOI:** 10.1111/cge.14763

**Published:** 2025-04-30

**Authors:** Maura Mingoia, Alessandra Meloni, Silvia Sedda, Sanaa Choufani, Isadora Asunis, Giorgia Gemma, Antonio Ammendola, Arteen Torabi‐Marashi, Eleonora di Venere, Gabriella Maria Squeo, Vincenzo Rallo, Maria Giuseppina Marini, Paolo Moi, Salvatore Savasta, Rosanna Weksberg, Giuseppe Merla, Andrea Angius

**Affiliations:** ^1^ Institute of Genetic and Biomedical Research, CNR Cagliari Monserrato Italy; ^2^ Antonio Cao Children's Hospital ASL Cagliari Italy; ^3^ Program in Genetics and Genome Biology, The Hospital for Sick Children Toronto Ontario Canada; ^4^ Department of Molecular Medicine and Medical Biotechnology University of Naples Federico II Naples Italy; ^5^ Laboratory of Regulatory and Functional Genomics Fondazione IRCCS Casa Sollievo Della Sofferenza, San Giovanni Rotondo Foggia Italy; ^6^ Department of Science and Technology University of Sannio Benevento Italy; ^7^ The Centre for Computational Medicine, The Hospital for Sick Children Toronto Ontario Canada

**Keywords:** alternative splicing, Au‐Kline, DNA methylation, HNRNPK, loss of function

## Abstract

Despite the massive adoption of sequencing technologies, disease‐specific diagnosis remains challenging, particularly for genes with highly homologous pseudogenes like *HNRNPK*. Pathogenic *HNRNPK* variants cause Au‐Kline syndrome (AKS), a neurodevelopmental disorder with malformations and distinctive facial features. We validated a novel *de novo HNRNPK* intronic variant (c.1192‐3 C>A, p.Leu398ValfsTer21) in a patient previously misdiagnosed with Kabuki Syndrome (KS). By combining sequencing, in vitro splicing assays, molecular modelling, and protein function analysis, we characterised the molecular defect. A unique DNA methylation (DNAm) signature was recently identified in AKS, with missense variants showing an intermediate DNAm pattern, suggesting an epi‐genotype–phenotype correlation linked to milder clinical features. The DNAm signature is a valuable tool for variant interpretation, especially in unclear AKS cases. We demonstrate that two independent approaches—functional characterisation and DNAm evaluation—confirmed a partial loss of HNRNPK function and validated an AKS diagnosis with a mild phenotype. Our findings highlight that a multidisciplinary approach integrating genomic and epigenomic analyses with functional studies and clinical assessment significantly improves rare disease diagnosis.

## Graphical abstract

Integrated genomic and epigenomic analyses, associated with functional studies, confirm the pathogenicity of a novel *HNRNPK* variant in Au‐Kline syndrome (AKS). Our study underscores the value of DNA methylation signatures in variant interpretation, enhancing accurate diagnosis and clinical management of rare neurodevelopmental disorders.

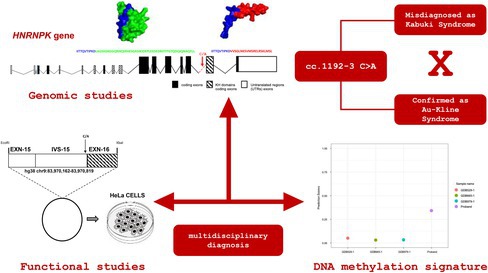

## 1 Introduction

Au‐Kline syndrome (AKS [MIM: 616580]) is a rare genetic disorder characterised by facial dysmorphisms, intellectual disability, developmental delays, skeletal abnormalities, and congenital heart disease [1]. Common features include feeding difficulties, visual and hearing impairments, osteopenia, craniosynostosis, and skeletal issues, while epilepsy and brain malformations are less frequent [1, 2, 3, 4].

AKS is caused by heterozygous loss‐of‐function (LoF) variants in the *HNRNPK* gene, which regulates gene expression and RNA transcription/translation [5, 6, 7]. Pathogenic variants were identified, with haploinsufficiency as the primary mechanism [1, 2, 3, 8, 9, 10, 11]. A specific *HNRNPK* DNA methylation pattern improved understanding of AKS variability and clarified missense variant effects [2, 11, 12].

HNRNPK regulates RNA molecules [6, 7] and long non‐coding RNAs, which can modulate clinical severity [13]. Pathogenic biallelic variants in other RNA‐binding genes, such as *RBM42*, also cause multisystem disorders resembling AKS, highlighting RNA regulation's role in genetic diseases [14]. Advances in transcriptome and exome analysis aid in diagnosing atypical AKS cases and identifying pathogenic *HNRNPK* variants [4, 15].

Here, we describe a Sardinian patient with a mild AKS form harbouring a novel *de novo* intronic *HNRNPK* variant. DNA methylation and functional studies demonstrated pathogenicity, leading to partial protein function loss.

## 2 Subjects and Methods

### 2.1 Clinical Report

**FIGURE 1:**
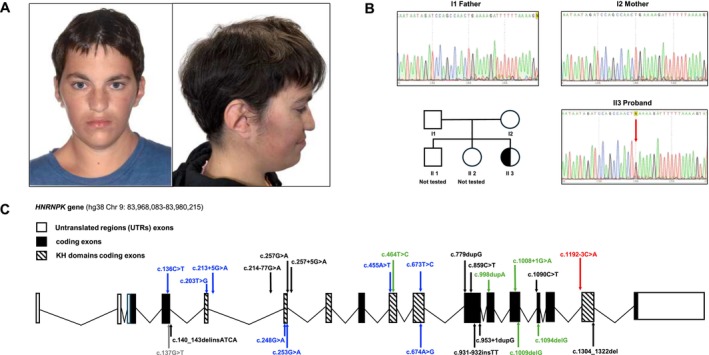
Clinical and genetical features. (A) Patient facial appearance. (B) Family tree and DNA electropherograms. Red arrow indicates C > A *de novo* variant. (C) Schematic representation of the *HNRNPK* gene. Variant positions based on NM_002140.4. Arrows indicate DNAm signature evaluation for AKS variants: Black, positive signature; Blue, intermediate signature; Grey, positive/intermediate signature; Green, clinically confirmed AKS, no signature; Red, our *de novo* intermediate signature; According to 2 and 11 references.

We investigated the clinical features of a Sardinian patient at the “Antonio Cao” Children's Hospital in Cagliari, presenting with neurodevelopmental delay and facial dysmorphism. Prenatal evaluation identified increased nuchal translucency, but chorionic villus sampling confirmed a normal female karyotype. Born at term with normal percentiles (Table S1), she exhibited talo‐valgus foot and hip dislocation, both surgically treated. Motor milestones were delayed, with autonomous walking achieved at 4 years. She developed aortic valve dysplasia with mild stenosis and tricuspid insufficiency. Features included dysmorphic traits, severe intellectual disability (IQ 40), and language difficulties. She attended school with special support and underwent speech and psychomotor therapy, achieving a moderate level in basic activities of daily living, such as toileting, hygiene, and feeding. At 12 years old, she was diagnosed with cervico‐dorso‐lumbar scoliosis, managed with a back brace until 16. A valgus deformity of the right elbow with radial head dislocation was noted. Puberty progressed normally, but the hot flashes led to endocrinological evaluation that confirmed a normal hormonal profile (Table S2). Hot flashes fit the autonomic dysfunction associated with AKS. Now 26 years old, she presents distinct facial features: arched eyebrows, elongated palpebral fissures (assessed through gestalt evaluation), broad nasal bridge, thick nasal alae, mandibular skewness, malocclusion, and dysmorphic ears (Figure 1A). Metabolic tests, EEG, VEP, and ABR were normal. Brain/spinal MRI revealed scaphocephaly and two small nonspecific white matter lesions. She also has dorso‐lumbar scoliosis, right arm extension limitation, and leg length discrepancy (1.5 cm). Autonomic dysfunctions include intestinal dysmotility, high pain threshold, and incomplete urinary control. Due to overlapping phenotypes, she was initially suspected of having Kabuki Syndrome (KS).

### 2.2 Multigene Panel Sequencing and Molecular Analysis

We extracted total DNA, RNA, and protein from PBMCs and buccal swabs. A customised NGS panel (HaloPlex target Enrichment) identified putative causative variants [16]. SpliceAI predicted splice site alterations caused by the mutant allele. The c.1192‐3 C>A was confirmed by Sanger sequencing. Protein expression was assessed using Western blotting.

### 2.3 In Vitro Analysis of Alternative Splicing

To validate SpliceAI predictions, a minigene was generated with exons 15–16 and intron 15 of *HNRNPK*, amplified with a Kozak sequence and a TAA stop codon, then cloned into pcDNA3.1. To quantify alternative splicing, transfected HeLa cells were analysed by RT‐qPCR.

### 2.4 DNA Methylation Array

DNA was bisulphite‐treated using the EZ DNA methylation kit. Methylation levels were quantified with Illumina Infinium MethylationEPIC v2.0 BeadChip and classified using EpigenCentral (http://epigen.ccm.sickkids.ca/).

### 2.5 Molecular Modelling

Splicing variant effects on protein domain structure were predicted in silico. HHpred identified the 3D NMR structure (379‐463 aa) of the HNRNPK, while AlphaFold modelled the mutated domain. Protein Data Bank (pdb1J5K) served as the template for mutation prediction. Pymol visualised wild‐type and mutated HNRNPK proteins.

### 2.6 Protein Functional Assay

Wt and mutant *HNRNPK* inserts were cloned into pEF5HA. Western blot confirmed protein expression in HeLa cells. Loss‐of‐function was verified by luciferase reporter assay. *ALOX15* 3' UTR region was amplified, cloned into pmiRGLO, and co‐transfected with *HNRNPK* vectors.

For a comprehensive description of the methodology refer to Additional File Data S1.

## 3 Results

We identified a novel heterozygous *HNRNPK* c.1192‐3 C>A variant (GRCh38; chr9:83970334; Figure 1B,C) associated with AKS. Classified as likely pathogenic (ACMG/AMP), its proximity to the intron 15/exon 16 splice junction and strong conservation (Figure S1A) suggested splicing disruption. SpliceAI predicted a new acceptor site (Δ score 0.83) (Table S3). RNA analysis was performed to confirm the alternative splicing event, but cDNA PCR and sequencing showed only canonical splicing (Figure S1B,C), probably because of high homology (96.1%) between *HNRNPK* and its pseudogenes *HNRNPKP2/HNRNPKP4*, which are indistinguishable from *HNRNPK* mRNA (Figure S1D).

**FIGURE 2:**
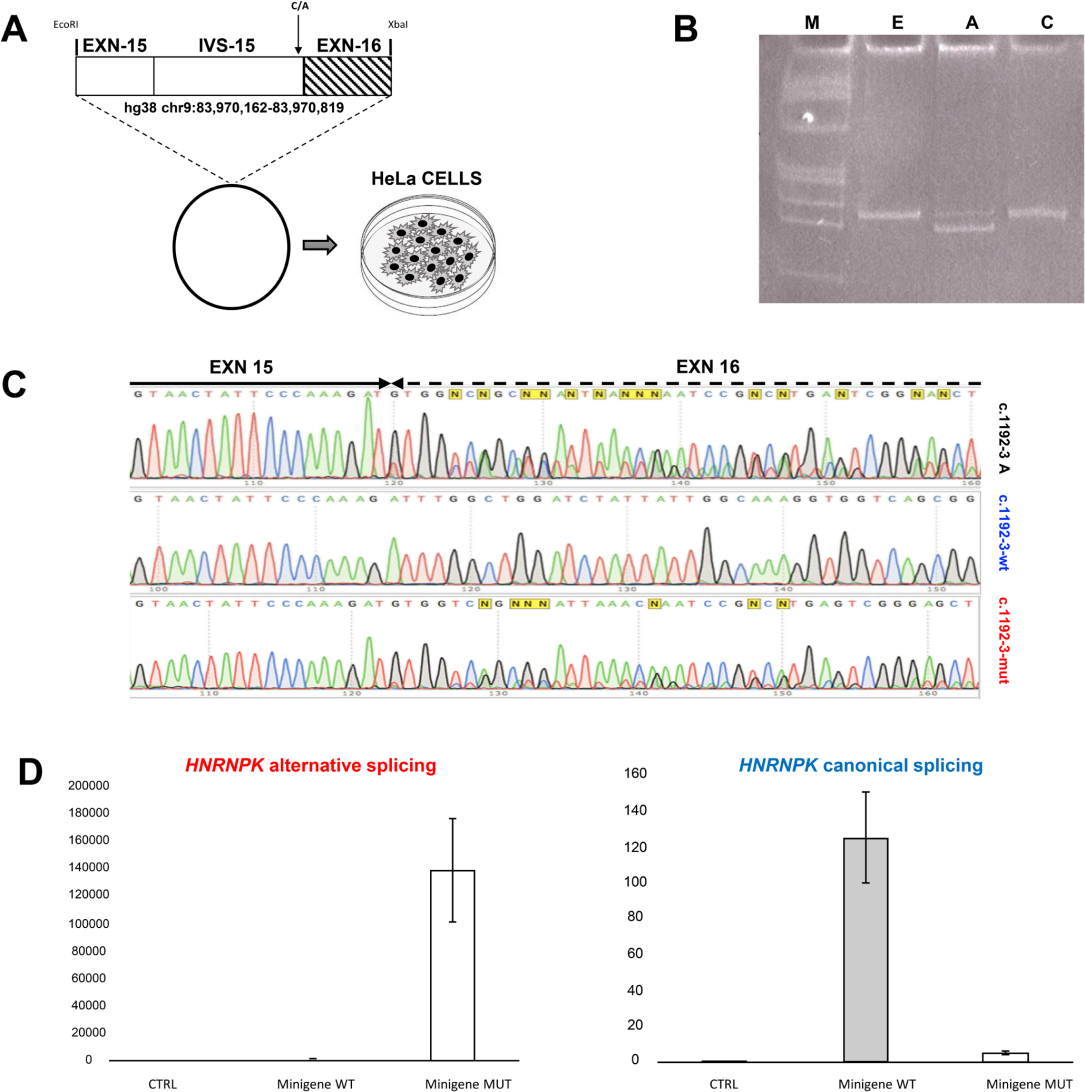
Analysis of alternative splicing. (A) Schematic representation of the synthesis of the *HNRNPK* minigene into the pcDNA3.1 vector. (B) Electrophoresis of the cDNA splicing event of the wt (lane C) and mutated (lane A) minigene. (C) Sanger sequencing of the *HNRNPK* minigene. Upper, splicing of minigene c.1192‐3 A; middle, canonical wt splicing; lower, mutated alternative splicing. (D) RTqPCR of the *HNRNPK*.

**FIGURE 3:**
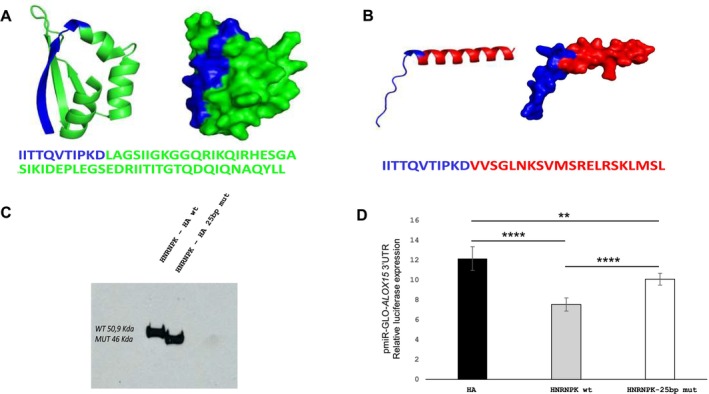
Modelling and functional analysis. (A) Three‐dimensional structure of wild‐type KH3 domain (387‐451aa) and (B) mutated (387‐397 aa + 21 mutated aa). (C) Western blot of wt and mutated HNRNPK protein in HeLa cells. (D) Luciferase 3'UTR expression of pmiR‐GLO‐*ALOX15* in HeLa cells.

HNRNPK protein isn't detectable by western blot in PBMCs or buccal cells, complicating protein analysis in accessible tissues (Figure S1E). To validate the presence of splicing, we generated a minigene with exon 15–16 and intron 15 by cloning wild‐type or mutant alleles into an expression plasmid and transfecting HeLa cells (Figure 2A). PCR revealed two fragments in mutant cells (Figure 2B) confirming by Sanger sequencing a 25‐bp frameshift deletion in exon 16, leading to a premature stop codon (p.Leu398ValfsTer21) (Figure 2C and S2). RT‐qPCR showed enrichment of mutant mRNA and reduced wild‐type expression (Figure 2D). Methylation profiling revealed an intermediate DNAm signature (SVM score = 0.34) (Figure S3). Samples with an SVM score > 0.25 were considered likely disease causing [11]. In silico analysis of the C‐terminus of HNRNPK (379–463 aa), using AlphaFold (pLDDT = 86.8, pTM = 0.345), showed the loss of one alpha‐helix and two beta‐sheets in KH3, impairing function (Figure 3A,B). The first 11 amino acids remained unchanged, but subsequent residues showed altered sequence and structure. To confirm the stability of the protein, the wt and mutant constructs were transfected into HeLa cells. Western blot showed that the mutant protein had a reduced molecular weight (50.9 kDa → 46 kDa) (Figure 3C). Functional impairment was assessed by luciferase assay using the 3'UTR of *ALOX15* [17] in pmiR‐GLO, which revealed reduced binding in mutant HNRNPK (Figure 3D).

## 4 Discussion

This study examines an individual with a phenotype overlapping with Kabuki syndrome (KS) and ultrarare Au‐Kline syndrome (< 40 cases). AKS, caused by *HNRNPK* mutations, has a distinct facial gestalt, but its expanding spectrum complicates diagnosis. We report a likely pathogenic *HNRNPK* splicing variant, though standard transcriptomic analyses showed no aberration, highlighting their limitations in detecting splicing defects in commonly studied tissues. To assess its impact, we applied genomic, epigenomic, and functional studies. *HNRNPK* pseudogenes complicate variant validation, mainly in PBMCs or buccal swabs, where HNRNPK was undetectable. Traditional methods may overlook splicing effects, requiring complementary approaches. We recommend minigenes for splicing analysis and molecular modelling for structural assessment. We confirm the variant induces aberrant splicing, leading to a partial loss‐of‐function (LoF). Luciferase assays revealed the truncated p.Leu398ValfsTer21 protein retains some functionality, likely due to intact KH1/KH2 domains compensating for KH3 loss [18]. Comparing our results with the *HNRNPK* DNA methylation (DNAm) signature [11] revealed an intermediate DNAm profile, aligning with milder AKS cases carrying KH‐coding missense variants. The lack of a full 3D protein model limits [19, 20] assessment of p.Leu398ValfsTer21's impact on global folding, emphasising the need for further structural studies. Rare *de novo* missense and intronic *HNRNPK* variants are frequently classified as VUSs, complicating AKS diagnosis. Integrating DNAm signatures with clinical assessment can refine AKS diagnosis and genotype–phenotype correlations. Our results confirm that the c.1192‐3 C>A variant causes HNRNPK truncation protein and partial LoF, underscoring KH3 domain's critical role. KH3 domain deletion severely impairs HNRNPK's RNA binding, disrupting key cellular functions linked to cognitive impairment. KH3 loss is pathogenic and highlighting this may aid in interpreting C‐terminal HNRNPK variants, enhancing AKS diagnosis. Since splicing defects may not be detectable in standard transcriptomic tissues, functional assays are essential for accurate variant interpretation. Future studies should explore HNRNPK interactions with target genes and tissue‐specific pathways to improve disease understanding and guide diagnostics.

## Conflicts of Interest

The authors declare no conflicts of interest.

## Peer Review

The peer review history for this article is available at https://www.webofscience.com/api/gateway/wos/peer‐review/10.1111/cge.14763.

## Supporting information


**Data S1.** Supporting Information.

## Data Availability

The data that support the findings of this study are available on request from the corresponding author. The data are not publicly available due to privacy or ethical restrictions.
