# Supplementary material for: A Novel Intronic Variant in the KH3 Domain of HNRNPK Leads to a Mild Form of Au‐Kline Syndrome
Source: Clin Genet. 2025 Apr 30;108(5):576–81. doi: 10.1111/cge.14763 (PMC12501752; doi:10.1111/cge.14763)

Supplementary Methods

Multigene panel sequencing, data analysis and molecular validation

We extracted total DNA, RNA and protein from parental and patient PBMCs and buccal swabs using standard methods. A customised HaloPlex target Enrichment NGS panel including about 67 known genes associated with chromatinopathies was applied accordingly to Squeo *et al*. to search for putative causing variants (1). Variants were classified using American College of Medical Genetics and Genomics (ACMG) guidelines (2). We used SpliceAI to predict acceptor or donor splice site alterations caused by the mutant allele (3). SpliceAI predicted the effects of alteration of acceptor and donor splicing sites with four Δ scores (Acceptor gain (AG), acceptor loss (AL), donor gain (DG), and donor loss (DL), quantifying the impact of variants relative to the reference allele (3). To confirm the presence of the c.1192-3 C>A variant and its segregation, genomic DNAs were Sanger sequenced using a 3130xl Genetic Analyzer (Applied Biosystem, Waltham, Massachusetts, USA) after PCR amplification with the following primers: 5'-ATGGTGATCTTGGTGGACCT-3'; 5'-TGCAGCAAATACTGTGCATTC-3'. To demonstrate alternative splicing of the HNRNPK coding transcript, the RNA was retrotranscribed using SuperScript III (Life Technologies, Carlsbad, California, USA) and the RT-PCR products were Sanger sequenced. Genome Browser (https://genome.ucsc.edu/) was used to assess the base wise conservation of HNRNPK at the c.1192-3 position towards vertebrates through PhyloP tool and to estimate genomic sequence homology in RT-PCR expected amplicon with Blat tool (https://genome.ucsc.edu/cgi-bin/hgBlat). To verify protein expression, a Western blotting assay was performed using 4-12% Bis/Tris NuPAGE gels (Life Technologies Carlsbad, California, USA). After blotting onto a PVDF membrane (Amersham, Buckinghamshire, United Kingdom), proteins were hybridised at room temperature with anti-HNRNPK, clone D6, and anti-β-actin, clone C4, antibodies (Santa Cruz Biotechnology, Dallas, Texas, USA) according to the manufacturer's protocol. After incubation with secondary antibodies, detection was performed using ECL Prime Western Blotting Detection Reagent and visualised using photographic film (Amersham, Buckinghamshire, United Kingdom).

Cell culture and transient transfection

HeLa and COS-7cells were grown in Dulbecco's modified Eagle's medium (Gibco, Thermo Fisher Scientific, Waltham, Massachusetts, USA) supplemented with 10% foetal bovine serum, 100 units/ml

penicillin – 100μg/ml streptomycin and 2mM glutamine (Euroclone, Milan, Italy) at 37°C and 5% CO_2. All transfections were performed using Lipofectamine 2000 transfectant agent according to the manufacturer's instructions (Life Technologies, Carlsbad, California, USA).

***In vitro* analysis of alternative splicing**

To assess the predicted alternative splicing by SpliceAI analysis, we created a minigene including the exon 15 – intron 15 – exon 16 of *HNRNPK* gene. Proband's DNA was amplified by using the 2xPhusion Master Mix (New England Biolabs, Ipswich, Massachusetts, USA) and the following primers:

5'-ATGAATTCGCCATGGATTCCTATGCAGGGTCGT-3' and

5'-ATATATCTAGATTACTGTTCTGCAGCAAATACTGTGCATTC-3'.

The forward primer included the restriction site for the EcoRI, a Kozak sequence and the ATG start codon. In the reverse primer the TAA stop codon triplet, and the XbaI restriction site were added at the 3' end. The resulting amplicon was verified by electrophoresis and then digested with EcoRI and XbaI restriction enzymes (New England Biolabs, Ipswich, Massachusetts, USA) for cloning into the pcDNA3.1 (Life Technologies, Carlsbad, California, USA) expression vector. Positive clones for the two minigene allelic versions [A mutant (mut) and C wild-type (wt)] were validated by Sanger sequencing. HeLa cells were transfected with 7.5 μg of the mutant, wt minigene and the empty pcDNA3.1 vector. 48 hours after transfection, cells were harvested for total RNA extraction using RNeasy kit (Qiagen, Hilden, Germany). The cDNA was synthetised with SuperScript III (Life technologies, Carlsbad, California, USA) and RT-PCR was performed. The resulting PCR products were Sanger sequenced to confirm the alternative splicing. To quantify the alternative splicing events, RTqPCR analysis with primers for canonical or alternative splicing was performed using ABI 7700 and 2X SYBR Green Master Mix (Applied Biosystems, Waltham, Massachusetts, USA). The results were normalised using endogenous *GAPDH* as an internal control.

cDNA obtained from K562 cells was amplified using primers carrying the sequences for the EcoRI and PmeI restriction enzymes recognition:

5'-ATGAATTCATGGAAACTGAACAGCCAGAAACCTT-3';

5'-TCGAAAGTTTAAACTTAGAAAACTTTCCAGAATACTGCTTCT-3'.

The mutant version was generated by site-specific mutagenesis using the Quick-Change II Mutagenesis Kit (Agilent Technologies, Santa Clara, California, USA) and the following primers:

5'-GTAACTATTCCCAAAGATGTGGTCAGCGGATTAAAC-3';

5'-GTTTAATCCGCTGACCACATCTTTGGGAATAGTTAC-3'.

After EcoRI and PmeI enzymatic digestion, the wt and mutant inserts were cloned into the pEF5HA expression vector (kindly provided by N. Bottini), which encodes for the haemagglutinin epitope as a tag. Positive clones for the two *HNRNPK* versions were screened by Sanger sequencing using 5'-ACACTGAGTGGGTGGAGAGACTG-3' primer.

Molecular modelling

Splicing variant effects on protein domain structure were predicted *in silico*. HHpred webtool (4) enabled the identification of the 3D NMR structure (379-463 aa) of HNRNPK protein, where KH3 domain is located (387-451 aa) on the UniProt database (5). The ColabFold protein folding platform, which implements AlphaFold (6), was utilised to create the model of the mutated protein domain (7). Prediction of the mutated structure was performed using the 1J5K pdb as template (8), obtained from the Protein Data Bank (9). Pymol (https://www.pymol.org/) was used to visualise and compare wt and mutated HNRNPK protein pattern.

Protein functional assay

HeLa and COS-7 cells were transfected with 10 μg of the pEF5-HA *HNRNPK* wt and pEF5HA *HNRNPK* mutant constructs. 48 hours post-transfection, cells were harvested and lysed for total protein collection. After SDS-PAGE separation using 4-12% Bis/Tris NuPAGE gels (Life Technologies, Carlsbad, California, USA), the proteins were blotted to a PVDF membrane (Amersham, Buckinghamshire, United Kingdom) and hybridised 1 hour at room temperature with 1:1000 anti-HA monoclonal antibody (Covance, Princeton, New Jersey, USA). Detection was performed using 1:20000 anti-mouse HRP-conjugated antibody (Santa Cruz Biotechnology, Dallas, Texas, USA) and ECL Prime Western Blotting Detection Reagent (Amersham, Buckinghamshire, United Kingdom).

To functionally assess the loss of function, we performed Luciferase assay. The 3'UTR region of the *ALOX15* was PCR amplified by using the primers carrying in 5' end XhoI and in 3' end SalI restriction sites:

5'-ATACTCGAGCGTCGCCACCCTTTGGTTATTTCA-3';

5'-GCCGGTCGACTTCTTGCCAATATTTTATTTAAAA-3'.

After enzymatic digestion and sequencing, the amplicon was cloned into the pmiRGLO vector (Promega, Madison, Wisconsin, USA), which includes the hRluc and Luc2 as reporter genes. HeLa cells were co-transfected with 1 μg pmiRGLO-*ALOX15* 3' UTR construct and 1,5 μg pEF5HA-empty or

pEF5HA-*HNRNPK* wt and mutated constructs. 48 hours after transfection, the cells were harvested and lysed. The luciferase expression assay was performed using the Dual Luciferase Reporter Assay Kit (Promega, Madison, Wisconsin, USA) and the Sinergy 2 luminometer (BioTek, Winooski, Vermont, USA). Renilla luciferase (hRluc) levels were normalised to luciferase (Luc2) luminescence activity for each sample, and all values were plotted relative to the pEF5HA empty vector.

DNA methylation array processing

Healthy controls and affected individual PBMCs DNA was bisulfite converted with the EZ DNA Methylation Kit (Zymo Research, Irvine, California, USA) following manufacturer's protocol. Bisulfite converted DNA was used for the analysis of whole genome methylation using the Illumina Infinium MethylationEPIC v2.0 BeadChip (EPIC v2, Illumina, San Diego, California, USA). Briefly, bisulfite converted DNA was whole-genome amplified for 20 hours followed by end-point fragmentation. Fragmented DNA was precipitated, denatured and hybridised to the BeadChips for 20 hours at 48°C. The BeadChips were washed, and the hybridised primers were extended and labelled before scanning by the Illumina NextSeq 550 system. Raw IDAT files were then shared with the Weksberg lab and classified using EpigenCentral (http://epigen.ccm.sickkids.ca/). IDAT files were uploaded to EpigenCentral along with a sample sheet detailing sample information. The data were classified on the HNRNPK signature with "Array Type" set to "Illumina HumanMethylationEPIC v2" and "Normalization Method" set to "Illumina".

Supplementary References

1. Squeo GM, Augello B, Massa V, Milani D, Colombo EA, Mazza T, et al. Customised next-generation sequencing multigene panel to screen a large cohort of individuals with chromatin-related disorder. J Med Genet. 2020 Nov;57(11):760–8.

2. Biesecker LG, Harrison SM, ClinGen Sequence Variant Interpretation Working Group. The ACMG/AMP reputable source criteria for the interpretation of sequence variants. Genet Med Off J Am Coll Med Genet. 2018 Dec;20(12):1687–8.

3. Jaganathan K, Kyriazopoulou Panagiotopoulou S, McRae JF, Darbandi SF, Knowles D, Li YI, et al. Predicting Splicing from Primary Sequence with Deep Learning. Cell. 2019 Jan;176(3):535-548.e24.

4. Gabler F, Nam S, Till S, Mirdita M, Steinegger M, Söding J, et al. Protein Sequence Analysis Using the MPI Bioinformatics Toolkit. Curr Protoc Bioinforma. 2020 Dec;72(1):e108.

5. The UniProt Consortium, Bateman A, Martin MJ, Orchard S, Magrane M, Ahmad S, et al. UniProt: the Universal Protein Knowledgebase in 2023. Nucleic Acids Res. 2023 Jan 6;51(D1):D523–31.

6. Jumper J, Evans R, Pritzel A, Green T, Figurnov M, Ronneberger O, et al. Highly accurate protein structure prediction with AlphaFold. Nature. 2021 Aug 26;596(7873):583–9.

7. Mirdita M, Schütze K, Moriwaki Y, Heo L, Ovchinnikov S, Steinegger M. ColabFold: making protein folding accessible to all. Nat Methods. 2022 Jun;19(6):679–82.

8. Braddock DT. Molecular basis of sequence-specific single-stranded DNA recognition by KH domains: solution structure of a complex between hnRNP K KH3 and single-stranded DNA. EMBO J. 2002 Jul 1;21(13):3476–85.

9. Berman HM. The Protein Data Bank. Nucleic Acids Res. 2000 Jan 1;28(1):235–42.

Table 1S.

Percentiles for weight, length, and head circumference at birth and in adulthood.

**Parameter**	**At Birth**	**In Adulthood (~20 years)**
**Weight**	3.43 kg (66th percentiles)	52 kg (15 th percentiles)
**Length / Height**	50 cm (68 th percentiles)	165 cm (P63 th percentiles)
**Head Circumference (HC)**	34 cm (54 th percentiles)	54 cm (P31 th percentiles)

Table 2S.

Assessment of the pituitary-gonadal axis at a three-week interval.

**Test**	**Date**	**Result**	**U.M.**	**Reference Range**
**FSH**	May 05, 2016	11.6	mU/ml	3.0–14.4 (Follicular phase)<br>5.8 - 21 (Mid - cycle peak)<br>1.2 - 9.0(Luteal phase)<br>< 3 (Prepubertal)<br>21.7 - 153 (Menopause)
	May 17, 2016	9.8		
	May 25, 2016	10.5		
**LH**	May 05, 2016	4.6	mU/ml	1.1 – 11.6 mU/ml (Follicular phase)<br>17 - 77 (Mid - cycle peak)<br>0 - 14.7(Luteal phase)<br>< 1 (Prepubertal)<br>11.3 – 39.8 (Menopause)
	May 17, 2016	7.8		
	May 25, 2016	10.7		
**Progesterone**	May 05, 2016	<0.20	ng/ml	0.33 – 1.2 ng/ml (Follicular phase)<br>0.72 – 17.8 (Luteinic phase)<br>0.27 – 0.9 (Adult male)<br>0.10 – 1.4 (Children)
	May 17, 2016	<0.20		
	May 25, 2016	7.13		
**Estradiol**	May 05, 2016	24.2	pg/ml	0 – 160 pg/ml (Follicular phase)<br>+/- 3 days 34-400 (Periovular phase)<br>27-246 (Luteinic phase)<br>0 – 30 (Menopause)<br>< 20(Prepubertal)
	May 17, 2016	148		
	May 25, 2016	57.4		

Table 3S.

SpliceAI analysis details.

SpliceAI scores: ⓘ

Variant	Gene (☐ = MANE Select transcript ☐ = non-coding transcript)	Δ type	Δ score ⓘ	position ⓘ
9:83970334-G-T	HNRNPK (ENSG00000165119.22 / ENST00000376263.8 / NM_031263.4)	Acceptor Loss	0.02	485 bp
⇒ 9:83970334 G>T	protein coding MANE Select transcript (minus strand)	Donor Loss	0.00	403 bp
UCSC, gnomAD	OMIM, GTEx, gnomAD, ClinGen, Ensembl, Decipher, GeneCards	Acceptor Gain	0.83	-28 bp
		Donor Gain	0.00	-409 bp

Figure 1S. Analysis of proband's mRNA.

A. Base wise conservation at the c.1192-3 position of *HNRNPK* toward multiple alignment of 8 vertebrates orthologous genes sequences using PhyloP tool in Genome Browser (https://genome.ucsc.edu/). Only zebrafish contain a different base at this locus (A vs G), confirming the strong conservation of the Guanosine in the other species considered; **B.** Agarose gel results of the RT-PCR product derived from Proband cDNA amplification for exon 15/16 of *HNRNPK*. Lane B: negative control; Lane M: DNA-ladder marker; Lane P: proband cDNA; Lane C: control cDNA. The amplicon size is 253 bp; **C.** Electropherogram of Sanger sequencing of Proband cDNA showing a canonical splicing junction between exon 15/16 of *HNRNPK*; **D.** Sequence homology calculation expressed in percentage of Identity using Blat tool in Genome Browser (https://genome.ucsc.edu/cgi-bin/hgBlat); **E.** Western blotting assay on protein extracts derived from parental and proband PBMCs and buccal swab cells. First and last lane: positive controls derived from Thp1 and HEK-293T cell lines.

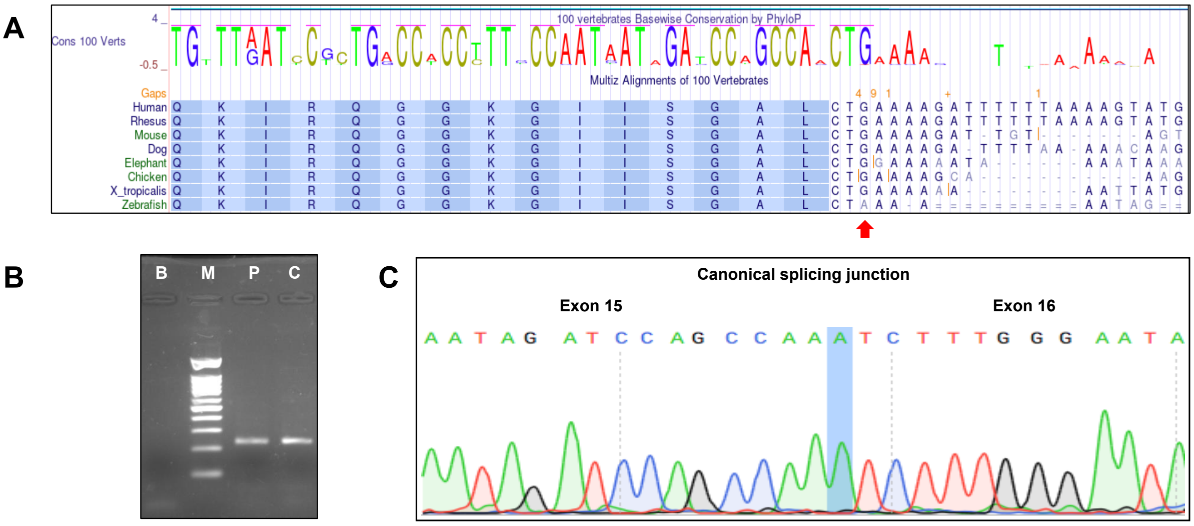

D

SCORE	START	END	QSIZE	IDENTITY	CHROM	STRAND	START	END	SPAN
252	1	253	253	100.0%	chr9	-	83970162	83970819	658
233	1	253	253	96.1%	chr2	-	136199145	136199397	253
233	1	253	253	96.1%	chr3	+	96350663	96350915	253
226	1	252	253	94.9%	chr5	-	127511491	127511742	252
193	1	253	253	86.8%	chr11	-	43262132	43262374	243
96	35	249	253	72.5%	chr1	+	85717596	85717810	215

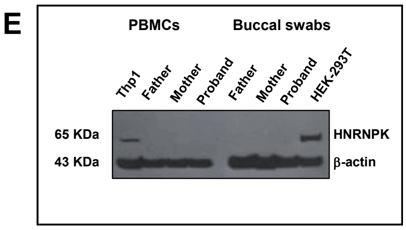

Figure 2S. Schematic representation of canonical splicing (in blue) and alternative splicing (in red) of intron 15 (in gray) in the presence of the c.1192-3 C or A variants.

Dashes indicate the loss of 25 bp in exon 16.

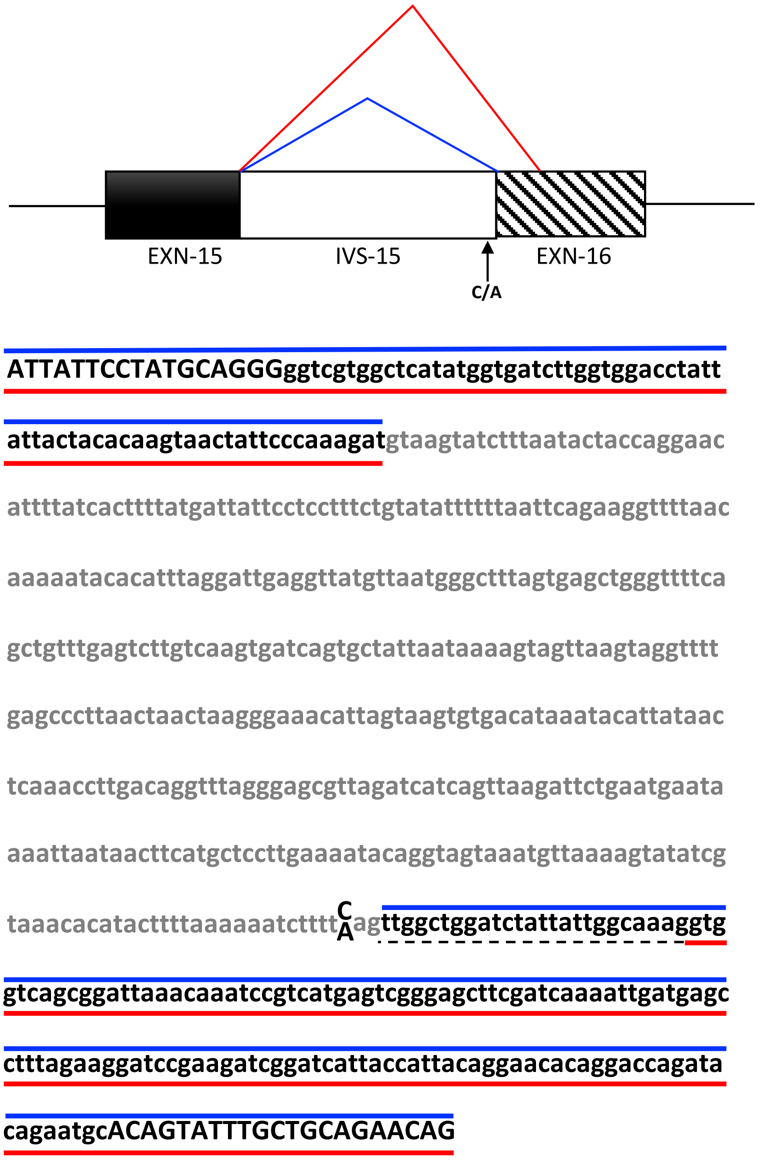

Figure 3S. Classification of variants using SVM machine learning models based on the *HNRNPK* DNAm signature.

Our Proband with the *HNRNPK* splice variant and three controls were classified according to the *HNRNPK* DNAm signature using the EpigenCentral web portal. Support Vector Machine (SVM) scores were generated and plotted for each individual using a previously described pipeline to generate scores using a disease-specific DNAm signature (Choufani S, et al. Am J Hum Genet. 2022). SVM scores range from 0 to 1, where 0 indicates a low probability of pathogenicity, while scores closer to 1 indicate a higher probability of pathogenicity. Samples with an SVM score> 0.25 were considered likely disease causing, while severe mutations identifying AKS high scores showed SVM score> 0.75.

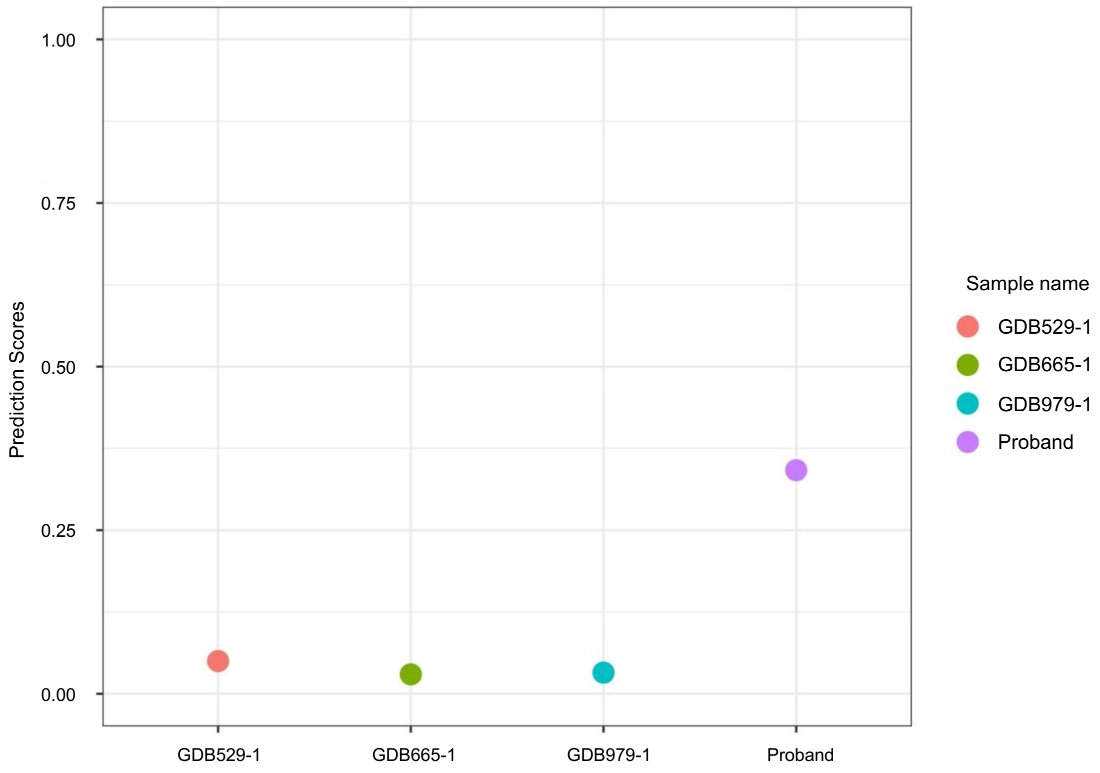

Supplement: Supplementary file 1 — Data S1. Supporting Information. [file CGE-108-576-s001.pdf]
